# 

**Published:** 1883-07

**Authors:** 


### *Bristol Medico-Chirurgical Journal.*

### JULY, 1883.

### CONTENTS.

ORIGINAL PAPERS :— *Page.*

The Proofs of the Existence of a Phthisical Contagion. By R. SHINGLETON SMITH, M.D., B.Sc., M.R.C.P. ... ... 1

Clinical Evidence against the Contagiousness of Phthisis. By E. MARKHAM SKERRITT, M.D. Lond., B.S., B.A., M.R.C.P. ... .. ... ... ... ... ... ... ... ... ... 48

The Cardiograph in Medicine. By G. MUNRO SMITH, L.R.C.P. Lond., M.R.C.S. ... ... ... ... ... ... ... ... ... 71

Surgical Out-Patient Notes. By W. H. HARSANT, F.R.C.S. ... 82

CLINICAL RECORDS :—

Two Cases of Compression of the Spinal Cord by Sarcomatous Growths from the Soft Membranes. By E. LONG FOX, M.D. Oxon., F.R.C.P. ... ... ... ... ... ... ... ... 100

A Case of Purulent Sub-Arachnitis, probably Metastatic. By GEORGE THOMPSON, M.D., L.R.C.P. ... ... ... ... ... 107

Cystic Neuroma of the Posterior Tibial Nerve. By J. FENTON EVANS, M.B. ... ... ... ... ... ... ... ... ... ... ... 109

Three Cases of Puerperal Convulsions treated by Venesection. By J. FULLER, M.K.Q.C.P. Irel. ... ... ... ... ... ... 111

Ulcerative Endocarditis of the Tricuspid Valve. Extensive Fibrinous Deposits. By J. E. SHAW, M.B. ... ... ... 114

Gastrostomy for Malignant Stricture of the Æsophagus. Hydatid Cyst of Liver. By the EDITOR ... ... ... ... ... ... 122

Recurrent Pendulous Fatty Tumour of Thigh. By the EDITOR 126

Primary Scirrhus of the Upper Jaw in a Child Two Years old 128

Case of Very Large Meningocele .. ... ... ... ... ... ... 129

Congenital Deformity of Right Leg and Left Hand ... ... ... 130

NOTES ON BOOKS ... ... ... ... ... ... ... ... ... ... ... ... 131

MEDICAL EXTRACTS ... ... ... ... ... ... ... ... ... ... ... 136

